# Suspiciousness perception in dynamic scenes: a comparison of CCTV operators and novices

**DOI:** 10.3389/fnhum.2013.00441

**Published:** 2013-08-22

**Authors:** Christina J. Howard, Tom Troscianko, Iain D. Gilchrist, Ardhendu Behera, David C. Hogg

**Affiliations:** ^1^Division of Psychology, Nottingham Trent UniversityNottingham, UK; ^2^School of Experimental Psychology, University of BristolBristol, UK; ^3^School of Computing, University of Leeds Leeds, UK

**Keywords:** eye movements, scene perception, expertise, security and human factors, visual search

## Abstract

Perception of scenes has typically been investigated by using static or simplified visual displays. How attention is used to perceive and evaluate dynamic, realistic scenes is more poorly understood, in part due to the problem of comparing eye fixations to moving stimuli across observers. When the task and stimulus is common across observers, consistent fixation location can indicate that that region has high goal-based relevance. Here we investigated these issues when an observer has a specific, and naturalistic, task: closed-circuit television (CCTV) monitoring. We concurrently recorded eye movements and ratings of perceived suspiciousness as different observers watched the same set of clips from real CCTV footage. Trained CCTV operators showed greater consistency in fixation location and greater consistency in suspiciousness judgements than untrained observers. Training appears to increase between-operators consistency by learning “knowing what to look for” in these scenes. We used a novel “Dynamic Area of Focus (DAF)” analysis to show that in CCTV monitoring there is a temporal relationship between eye movements and subsequent manual responses, as we have previously found for a sports video watching task. For trained CCTV operators and for untrained observers, manual responses were most highly related to between-observer eye position spread when a temporal lag was introduced between the fixation and response data. Several hundred milliseconds after between-observer eye positions became most similar, observers tended to push the joystick to indicate perceived suspiciousness. Conversely, several hundred milliseconds after between-observer eye positions became dissimilar, observers tended to rate suspiciousness as low. These data provide further support for this DAF method as an important tool for examining goal-directed fixation behavior when the stimulus is a real moving image.

## Introduction

Studies of naturalistic task performance have used eye movements as a measure of attentional deployment (e.g., Land, 1999; Findlay and Gilchrist, 2003; Underwood et al., 2003). Here we measure eye movements to investigate such attentional deployment in the context of closed-circuit television (CCTV) monitoring. CCTV monitoring is both a good model task in which to study the deployment of goal directed attention more generally and an important task more specifically because of its increased deployment in security and policing.

Recent research has examined human performance in some aspects of CCTV monitoring. For example, Troscianko et al. (2004) showed that people were able to anticipate antisocial behavior in the near future from CCTV footage. Others have examined the limitations of the use of CCTV footage in identifying unfamiliar individuals, although face recognition appears to be surprisingly resistant to viewpoint changes or poor image quality when individuals are familiar to observers (Bruce et al., 2001). However, much less is known about the dynamic allocation of attention during CCTV monitoring. Stainer et al. (2011) showed that eye movements in multiscreen displays tend to fall near the centres of individual video screens in a multiscreen display, and suggested that the lack of scene continuity and spatial contiguity between individual screens causes each one to be treated as an independent stimulus. However, there appears to be some direct competition between screens: Howard et al. (2011) showed that eye movements are driven to a great extent by the relative suspiciousness of different concurrent video screens in the display. CCTV is clearly a very rich visual stimulus and results are now beginning to emerge on several of the many aspects of human interaction with these stimuli. However, we are not aware of any work that seeks to examine exactly how attention is used within a single screen during on-line monitoring and decision making about video events, and this is addressed by the current study.

Examining how people perceive CCTV footage is one example of the more general task of perception of moving scenes. Much research has been conducted into the question of how we perceive static scenes and in particular, how long it takes to extract different types of visual information from scenes. Strikingly, the general “gist” of a scene can be processed from extremely brief (less than a tenth of a second) displays (e.g., Rousselet et al., 2005) or from a single glance (Biederman et al., 1974; Fei-Fei et al., 2007). When making global property classifications of a scene (e.g., naturalness, openness) and basic level categorisations (e.g., ocean, mountain), observers can reach asymptote levels of performance in 100 ms (Greene and Oliva, 2009). Little is known, however, about the time course of the perception of dynamic scenes. Of course outside of the laboratory, visual stimuli are rarely static and so it is important to investigate the extent to which the work with static images generalises to moving scenes. We recently examined this issue (Howard et al., 2010) by asking observers to make a continuous semantic judgement about a video of a semi-constrained real-world scenario: a football match. We found that responses continuously lagged behind eye movement behavior by over a second, suggesting that evaluation of moving scenes proceeds relatively slowly.

As well as being a task involving perception of real moving scenes, the task of monitoring CCTV images typically requires observers to search for and assess locations in the scene of maximum perceived suspiciousness. In this sense, whilst this task is very different from traditional visual search, some comparisons can be made. The task here could be considered as a visual search task for an extremely high level semantic target which is visually unspecified and could therefore take many different visual forms. From the traditional visual search literature, although target templates with high specificity are optimal for guiding attention, search can be driven by imprecise target information such as target categories (Malcolm and Henderson, 2009; Schmidt and Zelinsky, 2009; Yang and Zelinsky, 2009). Consistent with this, observers can use flexible target templates for search that are tolerant to some changes in target appearance e.g., changes in scale and orientation (Bravo and Farid, 2009, 2012). However, much less is known about the extent to which attention can be guided by very high-level semantic interpretation of scenes.

Another aspect of performance we addressed in the experiments presented here is the effect of expertise since our observers were both trained CCTV operators and untrained undergraduate observers. Howard et al. (2010) found that expertise affected the pattern of eye movements and the relationship between eye movements and responses. Specifically, individuals with more experience watching football matches made eye movements to goal relevant areas of the scene earlier than non-experts, and were thus able to spend longer evaluating the scene before making their responses. This would suggest that expertise may affect eye movement behavior in this CCTV monitoring task in a similar way i.e., that CCTV operators will be more able to direct their eye movements towards goal relevant areas of the scene than untrained observers. Indeed in a meta-analysis of several hundred effect sizes of expertise on eye movement behavior, Gegenfurtner et al. (2011) recently reported robust effects of expertise acting to increase frequency of fixations on goal relevant information and to reduce latencies for first fixations on the these areas. Some have claimed that this attention to goal relevant information (and consequently, reduced attention to irrelevant information) underlies the effect of expertise in visual tasks (Haider and Frensch, 1996). This “knowing what to look for” is likely in the CCTV operators since they are familiar with environments shown in the CCTV footage and the likely types of suspicious behaviors that may occur. For this reason, we hypothesised that CCTV operators would be able to process the scenes more efficiently than untrained observers. Scene “gist” or general layout can be extracted very rapidly but more detailed processing of scene content can take many seconds (e.g., Tatler et al., 2003). Given that CCTV monitoring requires complex semantic evaluation of scenes, we reasoned that this task would be likely to incur slower processing times and therefore may be sensitive to the effects of expertise.

There is evidence from a range of visual tasks that expertise affects processing efficiency. For example, in visual search tasks, domain-relevant expertise appears to enable observers to process a wider portion of their visual field at a time during visual search tasks (Hershler and Hochstein, 2009) and expert chess players can process visual information from across the visual field rapidly and with few fixations (Reingold et al., 2001). Similar processing advantages are seen in more applied tasks: expertise in driving facilitates wider visual scanning of road scenes (Underwood et al., 2002) and expertise in musical sight reading fosters greater storage of visual information from fixations on musical text (Furneaux and Land, 1999). We therefore hypothesised that CCTV operators would be able to process the unfolding CCTV scenes more efficiently than untrained observers. We expected CCTV operators to display greater between-observer consistency of gaze locations since their attention should be more consistently drawn to “suspiciousness” rather than other aspects of the scene or events within them.

We present here a task in which the attentional deployment of both trained operators and untrained, naïve observers is measured through eye tracking, whilst monitoring a single scene for potentially suspicious events. In this task, manual responses take the form of pushing a joystick to reflect the current degree to which events in the scene are perceived to be suspicious. The CCTV monitoring task requires continuous appraisal of the semantic content of scenes, and the evaluation of the current intentions and behaviors of people displayed in the scene. We will show a surprising degree of between-observer consistency in eye gaze locations in the scene, particularly between the gaze locations of trained CCTV operators. We will also show that periods of particularly high between-observer consistency in gaze positions are correlated with ratings of perceived suspiciousness in the scene. We will show that durations of between-observer eye position convergence are related to judgments of higher suspiciousness in the scenes, and that CCTV operators show longer periods of eye position convergence than untrained observers.

The current experiment demonstrates that this Dynamic Area of Focus (DAF) method works for a CCTV task as it did for the football watching task (Howard et al., 2010) with a different video stimulus and a very different semantic evaluation task. The DAF method is again shown to be a powerful tool for examining continuous perceptions of dynamic scenes without the need to analyse the content of the videos nor to measure low level physical characteristics or salience of the stimuli.

## Experiment 1: untrained observers

A computer programme was written in C++ to display a series of one–minute video clips of real CCTV footage obtained from Manchester City Council. Observers viewed a total of 40 one-minute clips comprising four clips from each of 10 different CCTV cameras. Observers viewed the videos in four blocks of ten minutes with breaks in-between, and the order of the 40 clips was quasi-randomised. The 10 cameras were chosen to represent as wide a range as possible in terms of the visual characteristics of the scenes. The ten camera views were as follows: night-time view of a carpark, pedestrian crossing, shopping street underpass, cash point at junction, busy retail street, landscaped open area, pedestrianised street, entrance to nightclub at night, bus stops and city centre street at night.

Observers made a constant judgement about the current perceived level of suspicious events in the scene by moving a joystick. Joystick ratings were sampled at 100 Hz resulting in a series of data points for each one-minute video stimulus. Note that this is a continuous response to a continuous stimulus, and the response takes the form of a rating about the video stimulus. Observers’ eye positions were recorded at 25 Hz throughout the task using the ASL Mobile Eye head-mounted eye-tracker and Eye Vision software.

Video clips measuring 27 degrees by 22 degrees of visual angle were projected in a dimly lit room against a white background using a Canon SX6 projector onto a screen at a distance of 1.6 m. Black chequerboard markers subtending 4 × 4 degrees were placed at each corner of the video display such that a computer algorithm could be used after data collection to stabilise eye position recording for changes in head position.

### Observers

Observers were thirty three undergraduate and postgraduate students at Bristol University, all naïve as to the purpose of the experiment, four of whom were male and twenty-nine female. The mean age of observers was 20 years, ranging from 18 to 34 years. All had normal or corrected-to-normal vision.

### Procedure

Observers were given written instructions as follows. They were asked to watch several videos of “urban scenes” and to monitor them for any suspicious events which, if seen in real life, might cause them to alert relevant authorities. They were asked to move a joystick according to what they perceived as being the current level of suspicious behavior in the video. They were told that at all times, the joystick should reflect what they perceived as being the current level of suspicious behavior. For instance, if they thought that the video was showing something very suspicious, they were told to move the joystick fully forwards for the duration of the suspicious events. If they perceived that there was currently absolutely no suspicious behavior, they were told not to move the joystick at all. They were informed that they could push the joystick to any level in-between these two extremes and that we would record the position of the joystick throughout the experiment. The joystick was in part chosen as a method of collecting responses as in the real CCTV control room where our operators were employed, operators may use a joystick to control the level of zoom on a particular camera, pushing the joystick further to zoom further in and vice versa. Hence this manual response was compatible with behaviors that occur in real CCTV monitoring contexts. Observers were asked to keep their hand on the joystick at all times to minimise the impact of manual reaction times.

## Experiment 2: trained CCTV operators

The method for Experiment 2 was similar to that used in Experiment 1, apart from the following differences. Video clips measuring 22 degrees by 18 degrees were projected against a white background. Observers viewed a total of 80 one-minute clips comprising eight clips from each of 10 different CCTV cameras. The 10 cameras were the same as those used in Experiment 1, but using twice as many clips from each: an extra four clips were used from each camera in addition to those used in Experiment 1. Observers viewed the videos in eight blocks of ten minutes with breaks in-between, and the order of the 80 clips was quasi-randomised. Observers completed the experiment in two sessions over the week long testing period.

### Observers

Observers were eleven trained CCTV operators working in the Manchester City Council CCTV control room of whom two were female and nine were male. All were naïve as to the purpose of the experiment but were aware that we were investigating the way that operators carry out their job, and the things that they look for whilst monitoring CCTV. All had normal or corrected-to-normal vision. The mean age of observers was 37 years, ranging from 23 to 60 years.

The trained CCTV operators differed from the untrained observers in terms of expertise since they had received training in the task of CCTV monitoring and all were currently employed as CCTV operators in the Manchester control room at the time of testing. Operators ranged in their level of experience from around six months to many years’ experience in the job. Different individuals undoubtedly had achieved differing levels of expertise in the task but on average these observers would certainly be more familiar with the task and the types of CCTV images used than the untrained observers.

## Results

### Ratings of perceived suspicious events

The mean suspiciousness rating across videos for the untrained observers was 0.397 and for the operators was 0.387 (0.417 for operators watching the 40 videos seen by both groups). There was no difference in the overall suspiciousness ratings given by the two groups (t(39) = 0.574, p = 0.57). The range of suspiciousness ratings across videos for the untrained observers was 2.769 and for the operators was 2.258 (2.313 for the operators when viewing those 40 videos also seen by the untrained observers). The untrained observers showed a greater range of ratings at each given time (between-observer variability in ratings) than the trained observers (t(39) = 3.16, p < 0.01). In other words, trained observers’ ratings were more consistent with one another at any given time.

### Consistency of eye gaze position

For each frame in each video stimulus, we calculated a measure of spread of eye positions. In an example frame, there will be one recorded eye position for each observer, each with a horizontal and vertical position. As a measure of spread in eye positions across observers at a particular time, we took the mean of the interquartile ranges of the horizontal and vertical eye positions. We used the interquartile range as a measure of variability to minimise the influence of position outliers. The subsequent “spread value” is a measure of the extent to which all observers were looking at the same part of the screen at the same time.

The mean spread measure, expressed as a fraction of the size of the display was 19.0% (SD = 3.0%) for the operators (18.8%, SD = 3.0%, for operators watching those 40 videos seen by both groups) and 22.0% (SD = 3.2%) for the untrained observers. Trained observers showed less eye position spread than untrained observers (t(39) = 6.07, p < 0.01) indicating that they were more likely to be looking at a similar point in the videos as one another at any particular time.

### Dynamic Area of Focus analysis: relationship between ratings and eye gaze position

The DAF analysis captures the relationship between moment-by-moment eye movement behavior and judgements of a group of observers viewing the same dynamic stimulus. To perform this analysis, we calculated estimates of the temporal relationship between eye movement behavior and responses. For these and all subsequent analyses, we calculated normalised suspiciousness ratings as follows: we first calculated the total overall mean and standard deviation of suspiciousness ratings for each observer and used these to normalise each observer’s data set. For each video stimulus, we then calculated for each frame, the median of the normalised ratings. We chose the median to minimise the effects of outliers in the data.

To calculate an estimate of the time lag between eye movements and responses, we performed correlations between eye position spread and these normalised manual responses. At each point in time for a particular video, there will be one value of between-observer eye position spread and one value of suspiciousness ratings across observers as defined above. Note that across each whole video, the manual responses and the eye movements are both time series data and hence do not represent a single point in time but rather a continuous stream of events that relate to the continuous video stimulus.

To test for a non-zero lag, we performed each correlation after artificially shifting the eye spread data forwards and backwards in time. For instance, to test for a 100 ms lag, we shifted the eye spread data 100 ms backwards in time relative to the response data and recalculated the correlation value. At the best estimate of the lag, this correlation should be maximally negative. The lag estimate is the estimated time delay between changes in eye movements spread and the manual responses associated with them. For example, a reduction in eye position spread might be associated with an increase in suspiciousness ratings a short while later, whilst an increase in eye position spread is likely to be associated with a decrease in suspiciousness ratings soon afterwards.

**Figure 1:**
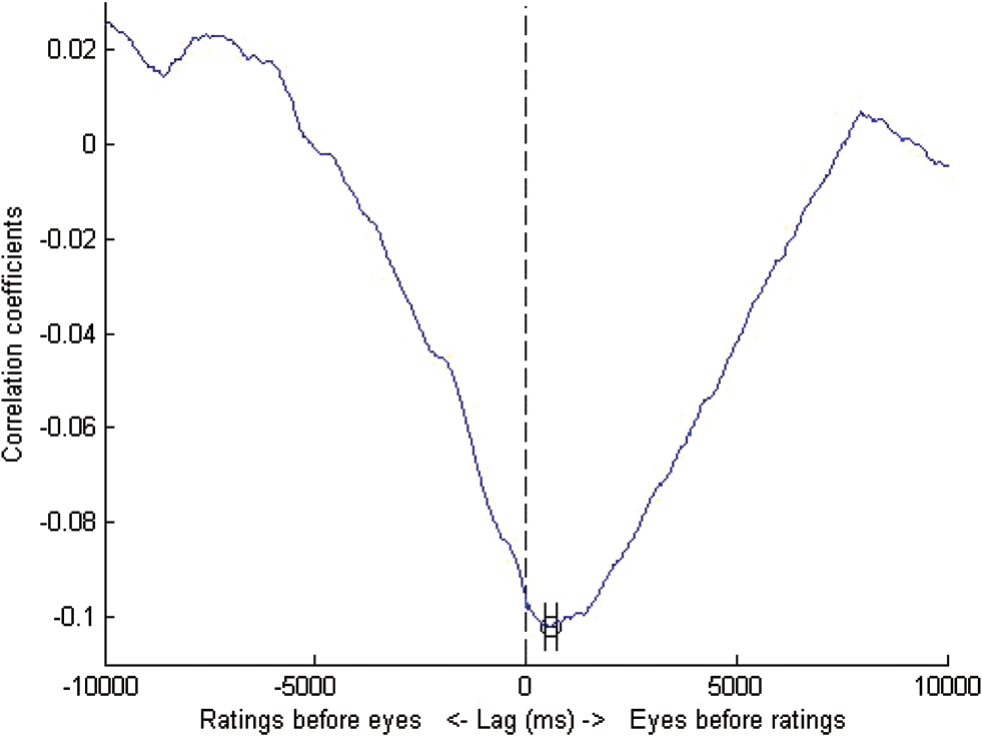
**DAF analysis for untrained observers**. The maximally negative correlation was obtained at a lag of 580 ms i.e., the eyes led manual responses by a lag of just over half a second. Error bars represent standard errors obtained by bootstrapping.

**Figure 2:**
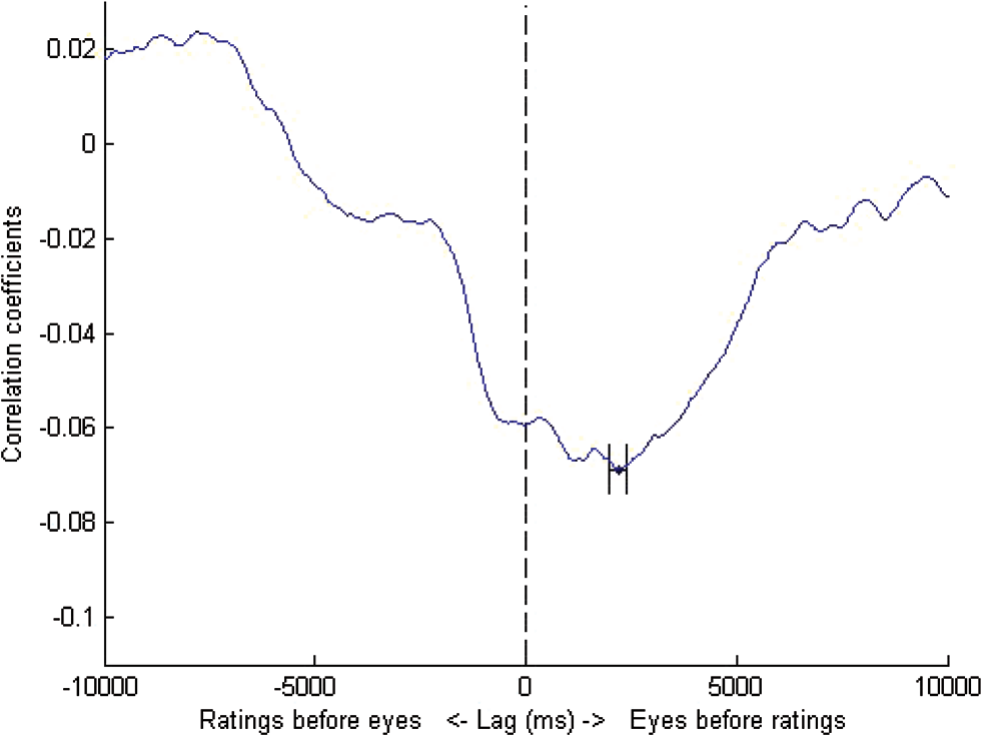
**DAF analysis for trained observers**. The maximally negative correlation was obtained at a lag of 2180 ms i.e., the eyes led manual responses by a lag of just over two seconds. Error bars represent standard errors obtained by bootstrapping.

Missing data created by these artificial time shifts were replaced with the mean value of spread for that stimulus. We tested each lag moving in steps of 10 ms through the range of up to 10 seconds both forwards and backwards in time. The results of these lag analyses are shown in Figures 1,2 below. Error bars were obtained by bootstrapping: we sampled observers (with replacement) to create bootstrapped “new” data sets and obtained the lag for each of these data sets. This bootstrapping cycle was repeated 50,000 times and the standard error of this set of lags was then calculated.

We reasoned that the spread values and ratings would be related to one another but not necessarily in a straightforward way. Two factors are likely to drive eye movements, namely goal relevance (in this case, suspiciousness) and also low-level image salience differences such as differences in brightness, colour and motion in the scene. In addition, there may be multiple areas of a scene for which either or both of these drivers attracts attention at any time. The extent to which observers will tend to fixate the same areas of the screen as one another (producing low spread values) was considered an empirical question. However, changes in eye position spread that occur close in time to changes in suspiciousness ratings may reflect scene events that are goal relevant. Of course, this is not to say that goal relevant events may not also be accompanied by changes in low-level scene salience, but by looking for the antecedents of high and low suspiciousness ratings, we will identify the extent to which eye position spread changes are related to goal relevance. We reasoned that if goal relevance is reliably related to eye position spread, then there will be a negative (and lagged) relationship between eye position spread and suspiciousness ratings. Overall, those events judged to be suspicious will tend to be preceded by different observers looking in similar places, and conversely, that events judged not to be suspicious will tend to be preceded by different observers looking in dissimilar places to one another. Since we are using a suspiciousness judgement along a continuum of joystick positions and not a discrete suspicious/not suspicious judgement, we must also consider that intermediate suspiciousness ratings will tend to be preceded by intermediate spread values, to an extent determined by how suspicious the scenes are judged to be.

Changes in eye position spread driven only by salience (for example, everyone’s eyes being drawn to a street light being turned on) will not be accompanied by a change in suspiciousness rating, and hence can only serve to decrease the strength of the correlation. Similarly, if there are two or multiple events in different parts of a scene that appear suspicious at any given time, this would produce high eye position spread measures and high suspiciousness ratings, thus decreasing the strength of the negative correlation between eye spread and ratings.

For both groups of observers at the obtained lags, eye position spread was negatively correlated with response (E1: r = −0.10, p < 0.05, E2: r = −0.07, p < 0.05) and these correlations coefficients between videos were significantly more negative than zero (E1: t(39) = −2.84, p < 0.01, E2: t(79) = −3.19, p < 0.01). The magnitude of the lag was much greater for the trained than the untrained observers. For those 40 videos seen by both sets of observers, the trained observers also showed a negative correlation between eye position spread and responses (r = −0.07, p < 0.01). This correlation was maximally negative at a lag of 1130 ms and was significantly more negative than zero (t(39) = −2.56, p = 0.01). Although this lag value is shorter than that seen when the data is analysed for the whole set of 80 videos seen by trained participants, it is still substantially longer than the lag found for untrained participants.

### Relationship between ratings and eye gaze convergence

**Figure 3:**
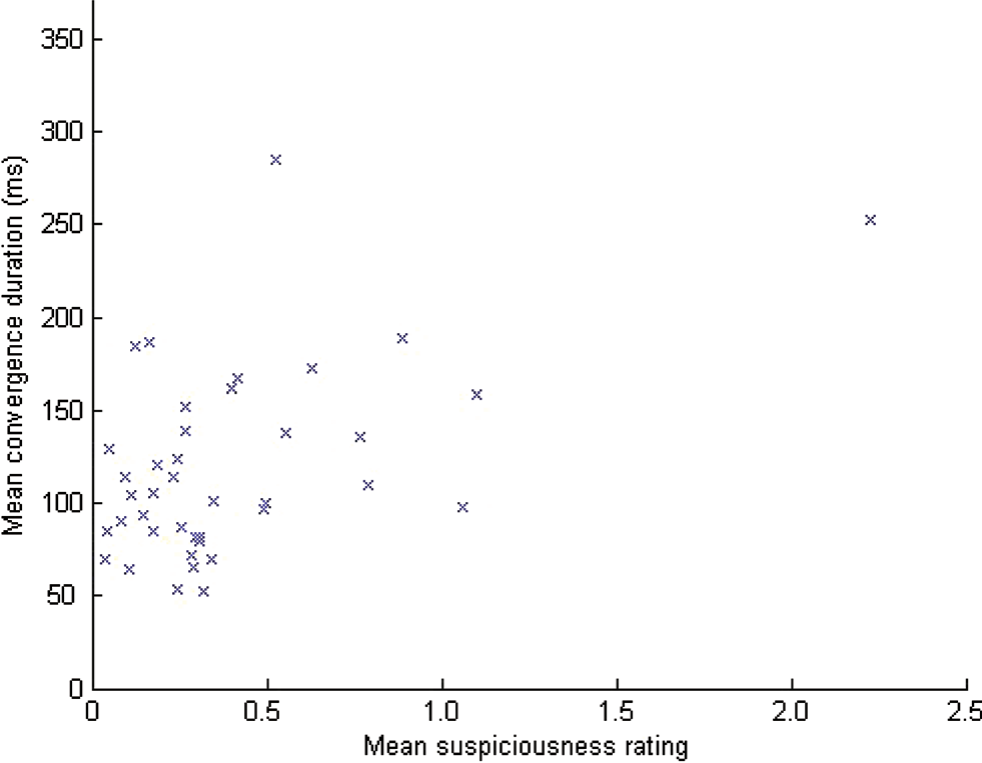
**The relationship between convergence period duration and ratings of perceived suspiciousness for untrained observers**.

For each frame in each video, we classed the spread measure as either “low” or “not low” using a threshold of one standard deviation below the overall mean spread value. We then calculated the durations of periods of time in which this thresholded spread value remained consistently “low” on subsequent frames. Overall for the untrained observers, the mean duration of these low spread (or equivalently, “convergence”) periods was 128 ms. For the untrained observers, there was a significant correlation (see Figure 3) between the mean convergence duration for each one minute video stimulus and the mean suspiciousness rating given to that video (r(39) = 0.509, p < 0.01). The data for one of the video stimuli was more than two standard deviations above the mean on both variables of mean convergence duration and mean suspiciousness rating, but the correlation remained significant even after excluding this data point (r(38) = 0.324, p = 0.044).

**Figure 4:**
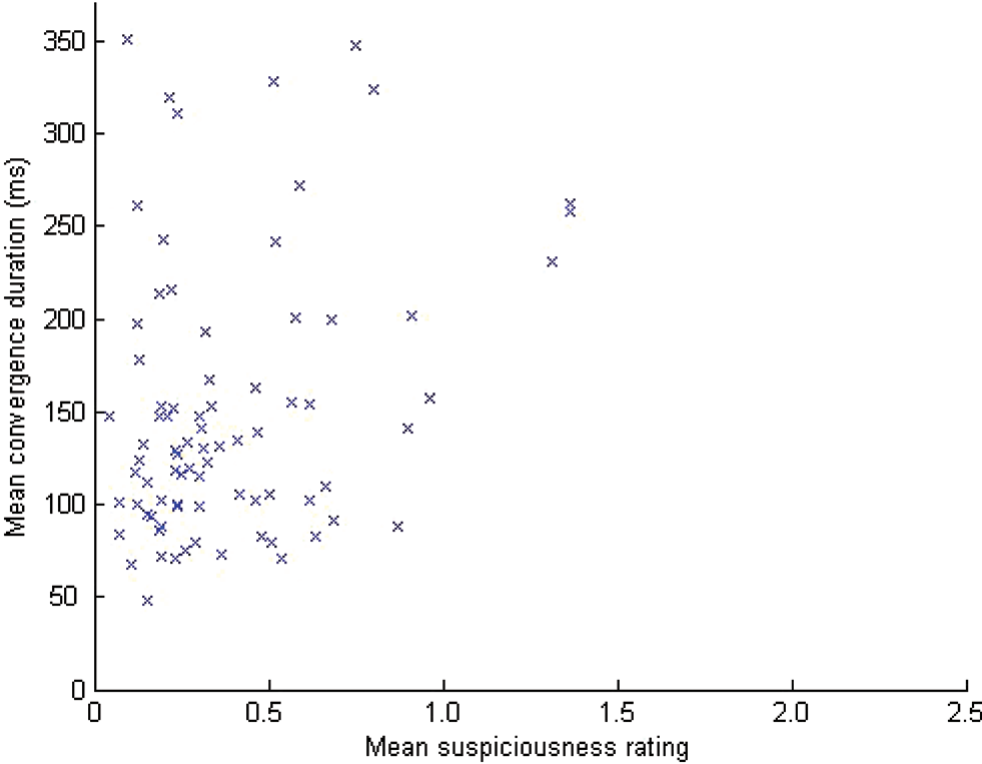
**The relationship between convergence period duration and ratings of perceived suspiciousness for trained CCTV operators**.

Overall for the trained CCTV operators, the mean duration of these low spread “convergence” periods was 151 ms. As shown in Figure 4, there was a significant correlation between the mean convergence duration and the mean suspiciousness rating for each one minute video stimulus (r(79) = 0.296, p < 0.01). For those 40 videos also seen by the untrained observers, there was also a significant correlation (r(39) = 0.356, p < 0.024).

There was no significant difference in the strength of correlations between trained and untrained observers (p > 0.05) for any of the correlations reported above (correlations between convergence duration and ratings). However, for the 40 videos seen by both sets of observers, mean convergence period durations were longer for the CCTV operators than the untrained observers (t(39) = 3.540, p < 0.01) indicating that CCTV operators were more likely to spend longer periods of time consistently looking at the same part of the screen as one another than was the case for the untrained observers.

## Discussion

For this complex task, the DAF analysis reveals a temporal relationship between eye movements and subsequent manual responses. These results indicate that as found previously for a sports monitoring task (Howard et al., 2010), this DAF method is a powerful one for examining eye movement behavior towards moving stimuli. The method circumvents the need to analyse events within the video, nor low-level physical properties of the stimulus in order to examine goal-directed attention.

We found a significant negative correlation between eye position spread data and manual responses. In other words, observers tended to push the joystick to indicate perceived suspiciousness at times shortly after between-observer eye position differences decreased, and tended to give low suspiciousness ratings shortly after eye position spread increased. The time lag between eye position spread changes and corresponding suspiciousness judgement responses is relatively long and in the order of hundreds of milliseconds to seconds. This lag between eye movements and corresponding suspiciousness responses was longer in CCTV operators than in untrained observers. CCTV operators show reduced between-observer eye position spread and longer periods of eye position convergence than untrained observers. They also showed a greater degree of between-observer consistency in terms of suspiciousness ratings. We also show a relationship between the mean durations of eye position convergence events and the suspiciousness assigned to different videos. As discussed above regarding the lag analysis between eye movement spread and responses, both salience and goal relevance are likely to drive eye movements to different extents depending on the nature of the events depicted at any given time. However, we find a relationship between eye position convergence duration and ratings. Hence, like eye position spread, eye position convergence duration appears to be a strong enough indicator of goal relevance to show up over and above any effects of salient but not task-irrelevant events or the effects of multiple simultaneous relevant events.

This task was simpler than that of monitoring many screens at once as is the case in real CCTV control rooms. However, for multiple screens, untrained observers are able to perform this task since their eye movements are driven by goal relevance (i.e., suspiciousness) to a much greater extent than they are influenced by low-level image properties (Howard et al., 2011). The current study shows that both trained and untrained observers are able to respond to a single screen in such a way that their eye movements are related to goal relevance. Therefore, it seems likely that trained CCTV operators would be able to perform multiple screen monitoring to the same level or to a superior extent than untrained individuals and this deserves future investigation. In real CCTV control rooms, operators will need to monitor very many screens at once for suspicious activity and this method provides a starting point for understanding such a complex task. One way in which this method captures some of the processes involved in real CCTV monitoring is the use of joystick pushing/pulling as the manual response since in the real control room a similar joystick is used for operators to zoom into areas of interest or suspicious activity. The use of real CCTV footage from the urban areas familiar to operators and a realistic suspiciousness judgement are also very close to the demands of real CCTV monitoring in the control room. For these reasons, there should be a good degree of generalisability from our findings here to real CCTV tasks in terms of the relationship between eye movements and manual responses.

The task of judging perceived suspiciousness was an inherently ambiguous one. For example, footage of individuals “loitering” in a car park at night may be judged as suspicious to a greater or lesser extent by different individuals depending on their interpretation of the events depicted. In fact, it is has been previously shown for the same task that mood state can alter these judgements whilst monitoring CCTV (Cooper et al., 2013) reinforcing the subjective nature of these judgements. Therefore, even for the trained CCTV operators, there can be no objectively “correct” rating. We did not attempt to provide a benchmark of “correct” responses for this reason, though anecdotally while watching the videos, higher ratings of suspiciousness were associated with behavior such as that mentioned above in a car park, similar “loitering” around the entrance to nightclubs after dark or in an urban shopping area pedestrian underpass. A formal analysis of the content of video that is judged to be more or less suspicious is possible though it is beyond the scope of the work presented here. One can identify those periods of time in different videos that were given the highest suspiciousness ratings, and locate areas of the screen that were fixated just before the ratings were given, allowing for the time lag between eye movements and ratings. Whilst the spread measure is relative in that it describes eye positions only in terms of how close they are to other observers’ fixations, one could identify the location of the centroid of these between-observer fixations to locate the most goal relevant areas of the screen whilst suspiciousness ratings are high. Characterising the content of such activity might be done either qualitatively by coding different behavior-environment interactions, or more quantitatively by looking for physical qualities of these video events.

The fact that analysis of video content is not necessary (though it is possible) for this technique makes this a powerful new tool for examining eye movements to dynamic scenes. One of the main challenges in studying eye movements to complex moving stimuli, is how to associate eye movements with different aspects of the stimuli, and therefore how to compare eye movements between observers. For simple stimuli such as a single target or a relatively small number of moving targets, dynamic areas of interest are a common method of analysis. One can use such a moving area to calculate fixations and dwell times etc., to particular stimuli of interest. However, this technique becomes unwieldy when there are very many targets, complex motion, shape changes, occlusion events or where stimuli are complex enough (such as in real-world scenes) that defining what is a target becomes non-trivial. There are additional problems with the use of dynamic areas of interest, such as how to define saccadic overshooting or undershooting, catch-up saccades and extrapolatory eye movements. The current technique circumvents all these problems.

We find that perception of dynamic scenes in this task proceeds relatively slowly: observers’ responses lag behind eye movement convergence by a minimum of several hundred milliseconds. This is considerably longer than the typical time periods required for rapid evaluations of static scenes such as the “gist” which can be extracted effectively in around 100 ms (e.g., Biederman et al., 1974; Rousselet et al., 2005; Fei-Fei et al., 2007). The task here was very different from typical gist perception studies in several respects. Typical gist perception studies present stimuli only for a limited time and often measure a threshold of simple scene judgements. Here, however, the judgement was continuous and required semantic processing beyond simple scene-type judgements. More complex perceptual representations of scenes have been studied in the context of encoding images into memory. For example, Tatler et al. (2003) showed that memory representations of gist formed very rapidly. However, other judgements about more detailed aspects of the scene, like shapes, colours and positions of the scene elements benefitted from very many more seconds exposure up to 10 seconds. From this and later similar findings (Melcher, 2006) one might assume that the time course of semantic scene perception is as slow as this. However it is entirely possible that the limit in these memory studies may have occurred only at the stage of encoding and not perceptual processing. The results of the current study indicate a slow time course for semantic perception of dynamic scenes that ranges from several hundred milliseconds to several seconds.

Our findings here are somewhat consistent with earlier findings for a similar task but with a different stimulus (Howard et al., 2010) where observers watched a real videotaped football match and made continuous judgements about imminent goal likelihood. In this sports evaluation task observers’ manual responses lagged behind gaze convergence by 1360 ms (non-experts) or 2260 ms (experts). The reason for the longer lags seen in the sports task than the CCTV task is not clear, but there are several differences between the two studies. The sports task is more constrained in terms of likely events. The CCTV task, by contrast, contains several different scenes, and several different types of events that are relevant to the suspiciousness judgement e.g., loitering in a car park, activity in a city shopping street, around a cashpoint etc. The CCTV task involved viewing ten different urban scenes with frequent changes between scenes. In contrast, the sports task stimulus was a single football match with a continuous shot from the same camera. Hence, the CCTV task contains more uncertainty in terms of what counts as the relevant perceptual variable, “suspiciousness”, than does the sports task where the relevant variable is “goal likelihood”. There may also be a greater social perception component inherent in the CCTV stimulus since the task involves making judgements about individuals’ intentions and interactions with one another. Nonetheless, some comparison can be made between the two tasks since both require continuous semantic evaluation of moving scenes and appear to incur processing delays over several hundred milliseconds.

Two factors are likely to make our estimates here for the time course of dynamic scene perception longer than that previously reported for static scene evaluations. First, our method includes the time it takes to prepare and execute a response to the visual stimulus. However, reaction times to produce a manual response to stimuli tend to be in the order of 200–250 ms (Goldstone, 1968; Green and von Gierke, 1984) and the magnitude of the lags here implicates additional contributing processes. Second, and most interestingly, the nature of the continuous task itself is likely to have caused these large time lags. Here we used a continuous video stimulus within which events unfold over time. One reason why perception of dynamic scenes may lag behind visual events is that the visual system often integrates information over a temporal window of at least 100 ms (e.g., Gorea, 1986; Watamaniuk and Sekuler, 1992) which is a physical necessity for information with a temporal component such as stimulus change or motion. Hence any temporal averaging may serve to increase these lags. Lags are also likely to be increased by the complex perceptual demands inherent in making decisions about these dynamic stimuli. For example, attending to the biological motion of humans and making judgements about their intentions is attentionally demanding and particularly important when the signal is degraded, ambiguous or subject to competition from other attentionally demanding stimuli (Thompson and Parasuraman, 2012). We also know that attending to multiple regions of a scene in terms of their visual features is attentionally demanding and can incur costs in terms of temporal lags (Howard and Holcombe, 2008; Lo et al., 2012). especially under conditions of competition for attention by different stimuli with similar features. In addition, whilst attending to video stimuli, one must use sustained attention, the nature of which is known to be different from that of transient attention (Ling and Carrasco, 2006) and it is possible that processing using sustained attention proceeds relatively slowly compared to the more transient attention that can be used for more briefly presented stimuli. Hence the complex and continuous nature of the stimulus and the task likely comprise a large component of the time course of this type of dynamic scene processing.

Howard et al. (2011) showed that observers could monitor four CCTV screens at once for suspicious events. Whilst this is very different from a traditional visual search task where the search array is typically static and the target is typically very well defined, these results and the results in the current study can be considered evidence that observers can perform visual search for extremely high level semantic targets. In the current study, this high level semantic target is “suspiciousness” which may take many different visual forms. This extends the literature from more traditional visual search tasks showing that observers need not be given complete or fully determined target template information to perform visual search in scenes (e.g., Malcolm and Henderson, 2009; Schmidt and Zelinsky, 2009; Bravo and Farid, 2012). In this task, for moving, complex scenes, attention can be guided by very high-level semantic interpretation of scenes.

Trained observers showed a greater lag between eye movements and manual responses than untrained observers. This is consistent with previous data for a similar task but when making judgements about a sports match (Howard et al., 2010). In the sports task, it appeared that expert observers were more able than non-experts to move their eyes to the goal relevant areas of the scene earlier, thus allowing them more time to produce their response. A similar mechanism could be operating here if CCTV operators “know what to look for” in the scenes. This would also explain the fact that CCTV operators showed greater between-observer consistency in eye position and longer periods of between-observer eye position convergence than our untrained observers. The fact that trained observers’ ratings were more similar to one another at any given time than was the case for the untrained observers is also consistent with this picture. It is worth noting that the CCTV operators were familiar with the scenes presented in the videos and hence their expertise may lie both in the task of CCTV monitoring itself and also in their specific knowledge of the scenes and environments depicted in the video footage. The CCTV operators’ reduced level of eye movement variability compared to untrained observers may account for why the relationship between eye position spread and responses was less highly correlated than it was for untrained observers. It might also help explain why the lag curve for expert observers was flatter and less pronounced than for untrained observers.

There is evidence that expertise affects visual processing efficiency in a range of different tasks. Hershler and Hochstein (2009) examined the influence of expertise during visual search. They found that experts in specific recognition of either categories of “cars” or “birds” appeared to be able to process visual information in their area of expertise from a wider portion of the search display with each fixation. This is consistent with an explanation of expertise on the grounds of a greater capacity for information processing across the spatial domain. These results are reminiscent of similar results in the vision-for-action literature. For example, Underwood et al. (2002) report that expert drivers scan a wider portion of the road scene than novices. In musical sight reading, Furneaux and Land (1999) find that experts and non-experts tended to look at positions in the musical text that are approximately one second ahead of the notes they are currently playing. However the expert musicians appeared to be able to store more information in this visual information memory buffer. In their information reduction hypothesis of skill acquisition, Haider and Frensch (1996) point towards selective attention to goal relevant information as the cause of improved performance in experts. This selection of task relevant information over non-relevant information could be operating here and elsewhere, though it is also possible that processing is more efficient even once the most critical areas of the scene are selected by attention. These two aspects of efficiency i.e., selection and post-selection processing, are difficult to tease apart in the data presented here. However, the effect of expertise in these very different types of tasks, visual search and vision for action, are likely to reside in efficiency of visual information processing, albeit at potentially different cognitive and perceptual stages.

Any visual processing efficiency differences may plausibly reduce the cognitive or mnemonic load for experts. Since the task presented here involves a high degree of perceptual, cognitive and mnemonic load, this may be especially beneficial to the experts in the current study. Cognitive complexity and memory load have been shown to influence fixation patterns with observers using fixations to regain goal relevant information under conditions of high load (Droll and Hayhoe, 2007; Hardiess et al., 2008). Hence experts may have needed to use fewer re-fixations in this manner, contributing to the difference in eye movement patterns observed between our two groups.

One further possibility for the locus of expertise in this and in our previously reported sports task (Howard et al., 2010) is that experts are better able to anticipate upcoming visual events. Some evidence that this may be the case is given by Didierjean and Marmèche (2005) who showed anticipatory representations in expert basketball players. This was evidenced by the fact that experts’ comparisons between pairs of gameplay configurations was poorer when making comparisons about pairs that moved forwards in time rather than backwards. It appeared that their representations had already moved the events on in time when presented with the future configuration. Perhaps the experts in the current study and in our previous sports monitoring task were more able to predict near future events and hence use their eye movements more efficiently.

At first glance it is not clear how these visual processing differences might account for the longer lags reported here for CCTV operators than untrained observers between eye movements and manual responses. However in the football task, experts appeared to move their eyes to the relevant parts of the scene earlier, and this could have been facilitated by superior visual processing. The longer lag between eye movement convergence and manual responses may be a result of experts deliberately adopting an accuracy-over-speed strategy, perhaps as a direct result of more confidence about making goal relevant fixations. Trained operators may choose to undertake more processing before reaching a decision about manual responses. Additional time observing events is likely to result in increased visual information and decreased ambiguity about events being displayed, and it is possible that operators use a waiting strategy to minimise the number of false alarms. Indeed in the real CCTV control room, operators use a similar type of joystick to zoom in to events in real time, zooming in and out as desired depending on the unfolding events. Zooming in to more closely examine a particular stimulus incurs some information cost since it narrows the field of view in that particular camera and carries the risk of missing events occurring at other locations in the scene. Hence experts may have learned to use a conservative criterion for making suspiciousness judgements. One factor to note here is that our two groups of observers differed along many dimensions including training and experience, knowledge and expectations of the scenes presented, gender, age, socio-economic background and specific instruction in the task. Of course any or all of these factors could have contributed to this difference.

In the CCTV task presented here, our experts were trained professional CCTV operators, compared to untrained psychology undergraduates. In the football task, all the observers were undergraduate psychology students, but they differed in their level of self-reported experience watching football matches. Hence, there was a greater difference in expertise level in this CCTV monitoring task than in the football task and this may account for some of the differences in the results. Other differences between the CCTV and football tasks include the greater level of constraint about events in the football match (i.e., events typical of a football match such as passes, tackles, goal attempts, etc.) than the CCTV task, which is video footage of several different types of urban scene. Additionally, the CCTV video is potentially much more of a task of social perception than the football task, since it requires judgements of intentions, potential future behavior and interactions between individuals. Therefore the data we present here are a second example of the successful application of this DAF method of measuring eye-hand lags in two very different contexts. The method enables the use of tasks with moving video stimuli from real-life scenarios, as well as on-line continuous judgements about these stimuli. We demonstrate that cognitive evaluation of these moving scenes is a somewhat slow process. The ramifications of this processing time when multiple screens must be monitored, as in CCTV monitoring, may be particularly severe.

## Conflict of interest statement

 The authors declare that the research was conducted in the absence of any commercial or financial relationships that could be construed as a potential conflict of interest.

## References

[B1] Biederman I. Rabinowitz J. C. Glass A. L. Stacy E. W. (1974). On the information extracted from a glance at a scene. J. Exp. Psychol. 103, 597–600 10.1037/h0037158 4448962

[B2] Bravo M. J. Farid H. (2009). The specificity of the search template. J. Vis. 9, 31–39 10.1167/9.1.34 19271904

[B3] Bravo M. J. Farid H. (2012). Task demands determine the specificity of the search template. Atten. Percept. Psychophys. 74, 124–131 10.3758/s13414-011-0224-5 22006527

[B4] Bruce V. Henderson Z. Newman C. Burton A. M. (2001). Matching identities of familiar and unfamiliar faces caught on CCTV images. J. Exp. Psychol. Appl. 7, 207–218 10.1037/1076-898x.7.3.207 11676099

[B5] Cooper R. Howard C. J. Attwood A. S. Stirland R. Rostant V. Renton L. (2013). Acutely induced anxiety increases negative interpretations of events in a closed-circuit television monitoring task. Cogn. Emot. 27, 273–282 10.1080/02699931.2012.704352 22780582

[B6] Didierjean A. Marmèche E. (2005). Anticipatory representation of visual basketball scenes by novice and expert players. Vis. Cogn. 12, 265–283. 10.1080/13506280444000021

[B7] Droll J. A. Hayhoe M. M. (2007). Trade-offs between gaze and working memory use. J. Exp. Psychol. Hum. Percept. Perform. 33, 1352–1365 10.1037/0096-1523.33.6.1352 18085948

[B8] Fei-Fei L. Iyer A. Koch C. Perona P. (2007). What do we perceive in a glance of a real-world scene? J. Vis. 7, 1–29 10.1167/7.1.10 17461678

[B9] Findlay J. M. Gilchrist I. D. (2003) Active Vision: The Psychology of Looking and Seeing. Oxford: Oxford University Press

[B10] Furneaux S. Land M. F. (1999). The effects of skill on the eye–hand span during musical sight-reading. Proc. Biol. Sci. 266, 2435–2440 10.1098/rspb.1999.0943 10643087 PMC1690464

[B11] Gegenfurtner A. Lehtinen E. Säljö R. (2011). Expertise differences in the comprehension of visualizations: a meta-analysis of eye-tracking research in professional domains. Educ. Psychol. Rev. 23, 523–552 10.1007/s10648-011-9174-7

[B12] Goldstone S. (1968). Reaction time to onset and termination of lights and sounds. Percept. Mot. Skills 27, 1023–1029 10.2466/pms.1968.27.3f.1023

[B13] Gorea A. (1986). Temporal integration characteristics in spatial frequency identification. Vision Res. 26, 511–515 10.1016/0042-6989(86)90194-x 3727416

[B14] Green D. M. von Gierke S. M. (1984). Visual and auditory choice reaction times. Acta Psychol. 55, 231–247 10.1016/0001-6918(84)90043-x 6464800

[B15] Greene M. R. Oliva A. (2009). The briefest of glances: the time course of natural scene understanding. Psychol. Sci. 20, 464–472 10.1111/j.1467-9280.2009.02316.x 19399976 PMC2742770

[B16] Haider H. Frensch P. A. (1996). The role of information reduction in skill acquisition. Cogn. Psychol. 30, 304–337 10.1006/cogp.1996.0009 8660787

[B17] Hardiess G. Gillner S. Mallot H. A. (2008). Head and eye movements and the role of memory limitations in a visual search paradigm. J. Vis. 8, 1–13 10.1167/8.1.7 18318610

[B18] Hershler O. Hochstein S. (2009). The importance of being expert: Top-down attentional control in visual search with photographs. Atten. Percept. Psychophys. 71, 1478–1486 10.3758/app.71.7.1478 19801608

[B19] Howard C. J. Holcombe A. O. (2008). Tracking the changing features of multiple objects: progressively poorer perceptual precision and progressively greater perceptual lag. Vision Res. 48, 1164–1180 10.1016/j.visres.2008.01.023 18359501

[B20] Howard C. J. Gilchrist I. D. Troscianko T. Behera A. Hogg D. C. (2011). Task relevance predicts gaze in videos of real moving scenes. Exp. Brain Res. 214, 131–137 10.1007/s00221-011-2812-y 21822674

[B21] Howard C. J. Troscianko T. Gilchrist I. D. (2010). Eye-response lags during a continuous monitoring task. Psychon. Bull. Rev. 17, 710–717 10.3758/pbr.17.5.710 21037171 PMC2971464

[B22] Land M. (1999) Motion and vision: why animals move their eyes. J. Comp. Physiol. 185, 341–352 10.1007/s003590050393 10555268

[B23] Ling S. Carrasco M. (2006) Sustained and transient covert attention enhance the signal via different contrast response functions. Vision Res. 46, 1210–1220 10.1016/j.visres.2005.05.008 16005931 PMC1557421

[B24] Lo S.-L. Howard C. J. Holcombe A. O. (2012). Feature-based attentional interference revealed in perceptual errors and lags. Vision Res. 63, 20–33 10.1016/j.visres.2012.04.021 22579792

[B25] Malcolm G. L. Henderson J. M. (2009). The effects of target template specificity on visual search in real-world scenes: evidence from eye movements. J. Vis. 9, 1–13 10.1167/9.11.8 20053071

[B26] Melcher D. (2006). Accumulation and persistence of memory for natural scenes. J. Vis. 6, 8–17 10.1167/6.1.2 16489855

[B27] Reingold E. M. Charness N. Pomplun M. Stampe D. M. (2001). Visual span in expert chess players: evidence from eye movements. Psychol. Sci. 12, 48–55 10.1111/1467-9280.00309 11294228

[B28] Rousselet G. A. Joubert O. R. Fabre-Thorpe M. (2005). How long to get to the gist of real-world natural scenes? Vis. Cogn. 12, 852–877 10.1080/13506280444000553

[B29] Schmidt J. Zelinsky G. J. (2009). Search guidance is proportional to the categorical specificity of a target cue. Q. J. Exp. Psychol. 62, 1904–1914 10.1080/17470210902853530 19459137

[B30] Stainer M. J. Tatler B. W. Scott-Brown K. (2011). “*Viewing multiplex displays:* effects of continuity of content and spatial contiguity on fixation selection”, in, Abstracts of the 16th European Conference on Eye Movements, Vol. 4, Journal of Eye Movement Research, eds Vitu F. Castet E. Goffart L., Marseille, 255

[B31] Tatler B. Gilchrist I. Rusted J. (2003). The time course of abstract visual representation. Perception 32, 579–593 10.1068/p3396 12854644

[B32] Thompson J. Parasuraman R. (2012). Attention, biological motion, and action recognition. Neuroimage 59, 4–13 10.1016/j.neuroimage.2011.05.044 21640836

[B33] Troscianko T. Holmes A. Stillman J. Mirmehdi M. Wright D. Wilson A. (2004). What happens next? The predictability of natural behaviour viewed through CCTV cameras. Perception 33, 87–101 10.1068/p3402 15035331

[B34] Underwood G. Chapman P. Bowden K. Crundall D. (2002). Visual search while driving: skill and awareness during inspection of the scene. Transp. Res. Part F. Traffic Psychol. Behav. 5, 87–97 10.1016/s1369-8478(02)00008-6

[B35] Underwood G. Chapman P. Brocklehurst N. Underwood J. Crundall D. (2003). Visual attention while driving: sequence of eye fixations made by experienced and novice drivers. Ergonomics 46, 629–646 10.1080/0014013031000090116 12745692

[B36] Watamaniuk S. Sekuler R. (1992). Temporal and spatial integration in dynamic random-dot stimuli. Vision Res. 32, 2341–2347 10.1016/0042-6989(92)90097-3 1288010

[B37] Yang H. Zelinsky G. J. (2009). Visual search is guided to categorically defined targets. Vision Res. 49, 2095–2103 10.1016/j.visres.2009.05.017 19500615 PMC2756560

